# Consolidating drug data on a global scale using Linked Data

**DOI:** 10.1186/s13326-016-0111-z

**Published:** 2017-01-21

**Authors:** Milos Jovanovik, Dimitar Trajanov

**Affiliations:** Faculty of Computer Science and Engineering, ’Ss. Cyril and Methodius’ University in Skopje, Rugjer Boshkovikj 16, P.O. Box 393, Skopje, 1000 Macedonia

**Keywords:** Methodology, Drugs, Drug products, Healthcare, Linked data, Open data, Data consolidation, Tools

## Abstract

**Background:**

Drug product data is available on the Web in a distributed fashion. The reasons lie within the regulatory domains, which exist on a national level. As a consequence, the drug data available on the Web are independently curated by national institutions from each country, leaving the data in varying languages, with a varying structure, granularity level and format, on different locations on the Web. Therefore, one of the main challenges in the realm of drug data is the consolidation and integration of large amounts of heterogeneous data into a comprehensive dataspace, for the purpose of developing data-driven applications. In recent years, the adoption of the Linked Data principles has enabled data publishers to provide structured data on the Web and contextually interlink them with other public datasets, effectively de-siloing them. Defining methodological guidelines and specialized tools for generating Linked Data in the drug domain, applicable on a global scale, is a crucial step to achieving the necessary levels of data consolidation and alignment needed for the development of a global dataset of drug product data. This dataset would then enable a myriad of new usage scenarios, which can, for instance, provide insight into the global availability of different drug categories in different parts of the world.

**Results:**

We developed a methodology and a set of tools which support the process of generating Linked Data in the drug domain. Using them, we generated the LinkedDrugs dataset by seamlessly transforming, consolidating and publishing high-quality, 5-star Linked Drug Data from twenty-three countries, containing over 248,000 drug products, over 99,000,000 RDF triples and over 278,000 links to generic drugs from the LOD Cloud. Using the linked nature of the dataset, we demonstrate its ability to support advanced usage scenarios in the drug domain.

**Conclusions:**

The process of generating the LinkedDrugs dataset demonstrates the applicability of the methodological guidelines and the supporting tools in transforming drug product data from various, independent and distributed sources, into a comprehensive Linked Drug Data dataset. The presented user-centric and analytical usage scenarios over the dataset show the advantages of having a de-siloed, consolidated and comprehensive dataspace of drug data available via the existing infrastructure of the Web.

## Background

Accessing comprehensive drug and healthcare data on the Web can be a challenging task. The main reason lies in the fact that the data is available in different formats, at distributed Web locations. Additionally, most of the drug and healthcare datasets are published for specific purposes only, so consequentially, they contain limited data. For instance, national drug repositories contain information about drug products which are approved and sold in the country [1–7]; websites aimed for the general public [8–13] contain descriptive drug use information, such as target, dosage, packaging and warnings; websites aimed for professionals [14–16] contain more specific drug data, such as active ingredients, chemical formulas, drug-drug interactions, food-drug interactions, toxicity, etc. Websites such as DrugBank [14, 17] contain more comprehensive drug data. However, the drug entries in their dataset are generic drugs, i.e. active ingredients of drugs, and not actual drug products which can be bought by patients. Various mobile and web applications [18–22] contain information about drug products, but the data they use is country-specific and is not available to the interested parties in an open format. Global drug product repositories exist as well [23–25], but they either do not provide means of getting the data in an open format, or are locked behind a pay-wall. Additionally, some of them provide data for drugs registered in countries from specific world regions only, or solely provide the name of the drug product in a specific country.

### The Linked Data approach

According to [26–29], the emergence of the Linked Data principles has introduced new ways to integrate and consolidate data from various and distributed sources. The Linked Data principles provide means and standards for representing, storing and retrieving data over the existing infrastructure of the Web. This enables publishing and contextual linking of data on the Web and with it, creating a Web of data, as opposed to the current Web of documents.

With this, the Web becomes a distributed network for standards-based data access, usable by software agents and machines. The interlinked nature of the distributed datasets provides new use-cases for the end-users, which are generally unavailable over isolated datasets. The Linked Data approach solves the issue of having ‘data silos’ in traditional relational database systems, silos which cannot link to other databases without a specific code written for the task, or specific mapping created in a data warehousing solution.

Additionally, the schema-flexibility of RDF provides means of independent data management and evolution, which is very well suited for the nature of the Web, but also for non-Web use in environments where data de-siloing and consolidation is needed [30, 31].

So far, as a result of the adoption of Linked Data principles by data publishers, the Linked Open Data (LOD) Cloud [32] has been populated with 1104 interlinked datasets [33]. These datasets, published on the Web following the Linked Data principles, belong to 8 different domains: government, publications, life sciences, user-generated content, cross-domain, media, geographic and social web.

### Motivation

Drug and healthcare data is already available on the Web, both as Linked Data in the LOD Cloud and as regular data on websites and in mobile applications, intended for human consumption. However, the drug data available currently as Linked Data is comprised of drug entities which are generic drugs, i.e. active ingredients of drugs, not actual drug products registered in a specific country, under a specific name, with a specific dosage form, strength and price, for which an end-user might be interested. Such end-users are the patients, the pharmacists, the doctors, but also medical institutions, pharmaceutical companies, etc, which need access to drug products from specific countries for a multitude of user-centric and analytical scenarios [34]: accessing general information about drugs which can be bought in a country, accessing information about the availability of different drugs and drug categories in different countries, accessing pricing information, etc.

The national drug data are generally available on the Web, but on regular webpages and not as Linked Data. In order to transform the data from the national drug registries into high-quality, 5-star Linked Data [35], we propose a set of methodological guidelines which aim to assist drug data publishers and other interested parties into generating a global Linked Drug Data dataset, consisting of official data about drugs registered for use in different countries. One such global Linked Drug Dataset would be accessible by using W3C standards via the existing Web infrastructure and would enable a myriad of new drug-analysis scenarios, including the above-mentioned ones. It would enable further data exploitations via development of innovative applications and services which use the data gathered from national drug registries of different countries in the world.

Such methodological guidelines should include steps aimed towards modeling and aligning the data, transforming the data into 5-star Linked Data, publishing the created datasets on the Web and defining use-cases or developing applications on top of the dataset. They should be aimed towards assisting drug data owners and publishers, such as the national governing bodies, medical institutions, pharmaceutical companies, pharmacies, etc., in publishing their data in the same aligned, Linked Data format. Their data, once transformed into Linked Drug Data and interlinked with the drug data already published using the same methodology, could be used for data-based analytics (by the medical institutions, pharmaceutical companies and governing bodies) or for reaching potential customers (by pharmacies).

In this paper, we propose a set of such methodological guidelines for consolidating drug data on a global scale, using Linked Data. In order to support our methodological guidelines, we have developed a set of open and publicly available tools, which can be reused in the process of applying the steps from the methodology over drug data from any country.

Using the proposed methodological guidelines and tools, we generated the LinkedDrugs dataset which consists of drug product data from twenty-three countries, and therefore validated the methodology. For this purpose we developed an automated system which gathers drug data from the official national drug registries of twenty-three different countries, executes data cleaning, aligns and transforms the data into 5-star Linked Data and publishes them on the Web in a common, aligned and consolidated Linked Data dataset. We then demonstrate a set of user-centric and analytical usage scenarios over the generated dataset, which are otherwise unavailable or very time-consuming in a scenario where a user works with the data available on the Web in HTML webpages.

### Related work

#### Linked Data projects in the healthcare domain

Numerous projects and efforts have already worked on transforming drug and healthcare data into Linked Data. According to [33], there are currently 83 life science datasets in the LOD Cloud. These datasets contain healthcare data from various subdomains, such as drugs, diseases, genes, interactions, clinical trials, enzymes, etc. The most notable are the Linking Open Drug Data (LODD) project, Bio2RDF and the Semantic Web Health Care and Life Sciences Interest Group at W3C.

The LODD project [36] focuses on interlinking data about drugs already existing on the Web, as described in [37, 38]. The data ranges from impact of the drugs on gene expression to results of clinical trials. The aim of the project is to enable answering of interesting scientific and business questions by interlinking previously separated data about drugs and healthcare. As part of their work, they have collected datasets with over 8,000,000 RDF triples, interlinked with more than 370,000 RDF links. However, it seems that the project has not been updated for some time now, and Bio2RDF has taken over the hosting and continual publication of the datasets.

Bio2RDF [39] is an open-source project which creates RDF datasets from various life science resources and databases, and interconnects them following the Linked Data principles into one comprehensive network [40–42]. The latest release of Bio2RDF contains around 11 billion triples which are part of 35 datasets [43]. These datasets hold various healthcare data: clinical trials (ClinicalTrials), drugs (DrugBank, LinkedSPL, NDC), diseases (Orphanet), bioactive compounds (ChEMBL), genes (GenAge, GenDR, GOA, HGNC, HomoloGene, MGD, NCBI Gene, OMIM, PharmGKB, SGD, WormBase), proteins (InterPro, iProClass, iRefIndex), gene-protein interactions (CTD), biomedical ontologies (BioPortal), side effects (SIDER), terminology (Resource Registry, MeSH, NCBI taxonomy), mathematical models of biological processes (BioModels), publications (PubMed), etc.

One of the datasets, which is a part of the LODD cloud and Bio2RDF, is the DrugBank dataset, described in [17]. It provides RDF data about drugs, such as chemical, pharmacological and pharmaceutical information, taken from an the existing DrugBank database [14, 17] of drug information. The DrugBank RDF dataset [44] contains over 766,000 RDF triples for 4,770 drugs. These drugs are generic drugs, i.e. active ingredients in drug products.

The World Wide Web Consortium (W3C) has established the Semantic Web for Health Care and Life Sciences Interest Group (HCLS IG) [45] to help organizations from the health domain in their adoption of the Semantic Web technologies. It is comprised of experts from around 30 W3C member organizations: research centers, universities, companies, health institutions, etc. Its mission is to develop and support the use of the technologies of the Semantic Web in the fields of healthcare, life sciences, clinical research and translational medicine [46]. It is comprised of various subgroups, which are focused on making the biomedical data available in RDF, developing and maintaining biomedical ontologies, etc.

In recent years, our research team gained experience in the drug and healthcare domain by applying the Linked Data principles and the Semantic Web technologies in the several different scenarios. We have transformed and published the drug product data from the Health Insurance Fund of Macedonia as 5-star Linked Data, by connecting it to the LODD and LOD Cloud datasets via the DrugBank dataset [47]. We have since extended this dataset with 5-star Linked Data about the Macedonian medical institutions and drug availability lists from pharmacies [48]. We have also used Linked Data for an analysis of the connections between drugs and their interactions with food, and recipes from different national cuisines, resulting in findings that uncovered the ingredients and cuisines most responsible for negative food-drug interactions in different parts of the world [49]. These projects helped us gain insight in the domain and identify the challenges of applying Linked Data in the domain.

#### Linked Data methodologies

There are a few methodologies defined in the Linked Data domain, which deal with the process of generating, publishing and using Linked Data. They are mainly focused on government data, but some are domain independent. The W3C Government Linked Data Working Group has created official guidelines for publishing and accessing open (government) data using the Linked Data principles [50], and with it they suggest three existing methodologies which can be used with the Linked (Government) Data lifecycle. These three methodologies are: (a) the methodology of Hyland et al., (b) the methodology of Hausenblas et al. and (c) the methodology of Villazón-Terrazas et al.

Hyland et al. [51] define a methodology for Linked Government Data, which consists of six steps. Their methodology is based on the specifications and best practices by the W3C, and consists of the following steps: (1) Identify, (2) Model, (3) Name, (4) Describe, (5) Convert, (6) Publish, and (7) Maintain. The methodology contains most steps which are part of the generally accepted Linked Data lifecycle, but is missing guidelines on how to use the generated Linked Data. The authors believe that the usage of the generated dataset should be left to the users and other interested parties, and according to them, is not a task for the Linked Data publisher.

Hausenblas et al. [52] state that the existing data management approaches - which assume control over the data, the schema and the data generation - cannot be used in the environment of the Web, due to its open and decentralized nature. Their methodology consist of the following steps: (1) Data awareness, (2) Modeling, (3) Publishing, (4) Discovery, (5) Integration, and (6) Use-cases. It also covers most of the general Linked Data lifecycle steps, but does not provide detailed guidelines for the process of publishing the generated Linked Data dataset on the Web.

Similarly to Hyland et al., based on their experience in linked government data production, Villazón-Terrazas et al. [53] define a set of methodological guidelines for generating, publishing and exploiting Linked Government Data. Their lifecycle consists of the following steps: (1) Specify, (2) Model, (3) Generate, (4) Publish, and (5) Exploit. This is the only existing methodology in the Linked Data domain which covers all of the lifecycle steps, but unfortunately is focused on government data.

In addition to these three methodologies selected by the W3C Government Linked Data Working Group, the LOD2 Project has developed an updated Linked Data lifecycle for extracting, creating, enriching, linking and maintaining Linked Data [54]. The Linked Data lifecycle supported by the LOD2 integrated environment consists of (1) Extraction, (2) Storage, (3) Authoring, (4) Interlinking, (5) Classification, (6) Quality, (7) Evolution/Repair, and (8) Search/Browsing/Exploration. Even though this is the only methodology which provides software tools for the denoted steps, and the number of steps here is larger than in the other methodologies, it still misses some key elements of the Linked Data lifecycle, such as the data modeling, the definition of the URI format for the entities and the ways of publishing the generated dataset. The provided tools are also general, and cannot be applied in a specific domain without further work and domain knowledge.

## Results

We developed a methodology and a set of supporting tools for the drug domain, which allowed us to streamline the incremental process of generating high-quality Linked Data of drug products from twenty-three countries. This newly created dataset of drug product data, the LinkedDrugs dataset [55], currently consists of over 248,000 drug products, interlinked with over 91,000,000 relations denoting similarity between them, and with over 278,000 links to their corresponding active ingredients available as Linked Data in the LOD Cloud. The LinkedDrugs dataset enables a novel way of using drug product data, by unlocking new user-centric and analytical usage scenarios, previously unavailable over isolated and siloed drug data repositories. These scenarios utilize the consolidated and aligned nature of the dataset and its contextual links to entities from the LOD Cloud. This is achieved by automatically generating the additional relations in our dataset which link the drugs between themselves, and link the drugs with drug entities and active ingredients published as part of other LOD Cloud datasets. Using W3C standards over the existing infrastructure of the Web, we are then able to retrive data from these distributed datasets, and present them to the end-users in a comprehensive manner.

Here, we will provide a brief overview of the developed methodological guidelines and their tools aimed at assisting the data publishers during the specific steps in the methodology, while a more in-depth description is provided in the ‘Methods’ section. We will then present the LinkedDrugs dataset, along with the process of developing the automated system which constructs the dataset. Then, we will present an overview of newly enabled usage scenarios over the consolidated drug product data.

### The methodological guidelines

Our methodological guidelines for consolidating drug data using the Linked Data approach improve upon the existing Linked Data methodologies and contain steps, activities and tools which are specific to the drug data domain. We used our experience in the domain of Linked Healthcare Data to develop guidelines which aim to guide data publishers through the process of generating high quality, 5-star Linked Data in order to interlink, align and consolidate drug data from different national drug registries or other sources of drug data. The alignment and relationship between the existing methodologies and our guidelines is outlined in Table 5.

**Fig. 1:**
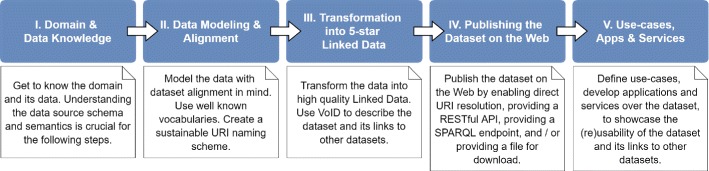
The methodology for consolidating drug data using the Linked Data approach

Our methodology consists of five steps (Fig. 1): (1) Domain and Data Knowledge, (2) Data Modeling and Alignment, (3) Transformation into 5-star Linked Data, (4) Publishing the Linked Data Dataset on the Web, and (5) Use-cases, Applications and Services. These steps have been developed with reuse as a primary goal; therefore, their main focus is the encouragement of data publishers in the drug domain to develop, modify and use reusable components during the steps of the methodology. This makes the Linked Drug Data lifecycle modular, i.e. constructed of loosely-coupled components which can be reused in the domain. Here, by loosely-coupled we mean components which can be used separately when necessary, but which also form a seamless workflow for generating a high-quality, 5-star Linked Drug Data dataset. The reuse of such components reduces development time and increases productivity [56, 57].

### Methodology supporting tools

As part of the methodological guidelines, we developed a set of tools as reusable components which simplify the execution of the specific steps of the methodology. Their intent is to support the Linked Drug Data generation process for both people from the drug domain which do not have deeper knowledge of Linked Data, and Linked Data publishers which do not have deeper knowledge of the drug domain. The set consists of (a) the RDF schema, (b) the CSV template, (c) the OpenRefine transformation script, (d) the SPARQL-based tool for extending and interlinking the dataset and (e) the web-based tool for automated transformation, interlinking and publishing of the generated Linked Drug Data dataset. These reusable components are open and publicly available on GitHub [58].

**Fig. 2:**
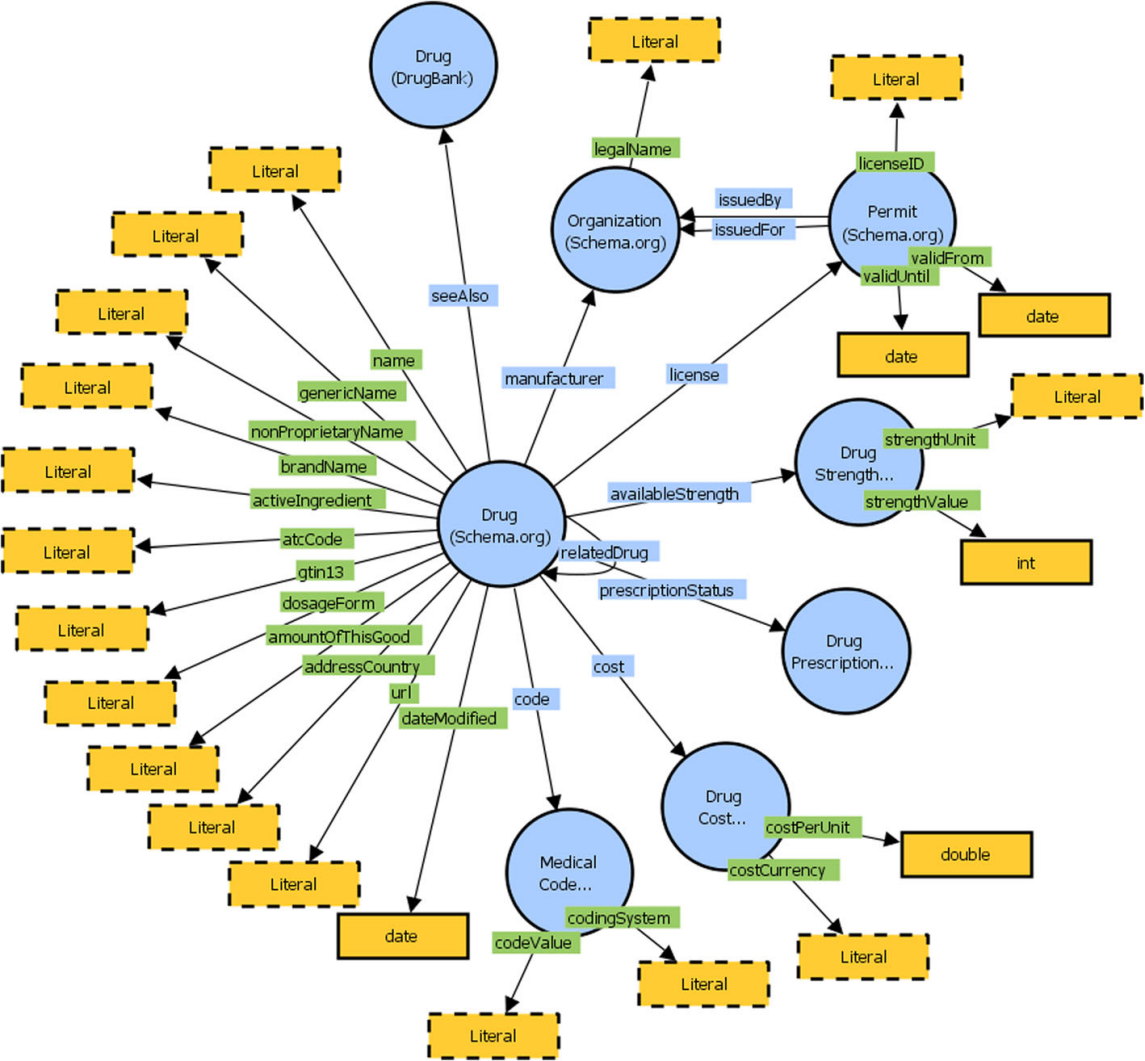
The RDF vocabulary designed for the drug data domain, comprised of Schema.org classes and properties. For dataset interoperability, it also uses the classes from the ATC Classification Ontology and properties from the DrugBank Ontology and RDFS

The RDF schema is a common data schema for all national drug data repositories, used for modeling of drug products on a global scale (Fig. 2). Its goal is to provide alignment of drug data from different sources, with different format and different levels of data granularity, in order to enable simpler data exploitation. It is comprised of classes and properties from the Schema.org vocabulary [59], which is a novel approach in the drug data domain.

The CSV template represents the necessary formal template for the data which is being prepared for transformation, i.e. the data which will be annotated with our RDF schema. It contains 39 columns which represent the different data fields needed from the source data for the transformation process. They inlude the URI of the drug, its brand name, generic name(s), manufacturer, ATC code(s), active ingredient(s), strength, cost, license information, etc. They are modelled to fit the RDF schema, which encompasses all data necessary for high-quality modeling of the domain.

The OpenRefine transformation script is a reusable tool which helps automate the transformation process, while ensuring compliance of the generated data with the defined RDF schema and therefore provides aligned, high-quality 5-star Linked Data for the drug domain. Its intent is to lower the bounds of transforming data into RDF and Linked Data for data publishers which are not deeply involved and experienced in the Semantic Web and Linked Data practices. Through several sub tasks, this reusable script for the OpenRefine-based suite of tools (e.g. LODRefine, BatchRefine), interlinks the drug products between themselves based on the therapeutic, pharmacological and chemical properties of the drugs, links the drug products to generic drugs, i.e. active ingredients from the LOD Cloud datasets and transforms the data into RDF format.

The SPARQL-based tool for extending and interlinking the dataset is comprised of two reusable SPARQL queries. The first query extends the dataset by assigning ATC codes to all drugs from the dataset which miss this information. It does so by using the interlinked nature of the dataset, i.e. the links the drug products have to generic drugs and active ingredients from the LOD Cloud. The second query detects all pairs of drugs from the dataset which have the same ATC code, and then interlinks them with properties from the RDF schema which denote the therapeutic, pharmacological and chemical similarity of the drugs.

The web-based tool for automated transformation, interlinking and publishing of the generated Linked Drug Data dataset is intended for data publishers which generate Linked Drug Data with the previous tools. The data publishers can upload the generated Linked Data dataset(s) on the LinkedDrugs project website [60], and after a human-based quality assessment, the dataset will be automatically published. For this we use a publicly available Virtuoso instance [61], from which the new dataset is available on the Web as Linked Data, via its SPARQL endpoint [62].

### LinkedDrugs: global linked drug products dataset

We applied the outlined steps of the methodology and the set of tools as part of an automated system for transforming and generating 5-star Linked Drug Data from twenty-three countries: Austria, Azerbaijan, Costa Rica, Cyprus, Egypt, Estonia, Ireland, Macedonia, Malta, Netherlands, New Zealand, Nigeria, Norway, Romania, Russia, Serbia, Slovakia, Slovenia, South African Republic, Spain, Uganda, Ukraine and USA. The countries were chosen to represent the global diversity and to show that a holistic solution for generating Linked Drug Data on a global scale is possible. Currently, the generated LinkedDrugs dataset contains over 99,000,000 RDF triples, which represent data for over 248,000 drug products from the denoted countries. The dataset also contains over 91,000,000 schema:relatedDrug relations between the drugs, and over 278,000 rdfs:seeAlso relations to generic drugs from DrugBank and DBpedia.

This automated system and its workflow represent a concrete example of applying the methodological guidelines and supporting tools presented in this paper, and thus serve as their validation scenario.

#### Generating the LinkedDrugs dataset

The national drug registries of many countries around the world are available online. We analyzed the national drug registry websites of 31 countries in order to define a common set of properties, i.e. a schema skeleton, for the target dataset. These steps of domain analysis and RDF schema definition correspond to the activities denoted in Step I and Step II of the methodological guidelines, which are already done and we directly use them for our specific application.

In order to design, test and validate our automated system for gathering 2-star drug data from the national drug registries and generating 5-star Linked Data from the drug domain on a global scale, we selected a subset of twenty-three countries. We aimed for a diverse subset, which will encompass different global regions.

The drug registries of these countries are available online. Their websites are listed in the project page on GitHub [58]. The drug data from most of the national registries is available in a structured format in HTML pages, intended for human consumption via searching and browsing on the website itself. The data from a smaller group of countries is available via structured files in Microsoft Excel or PDF formats, available for direct download.

**Fig. 3:**
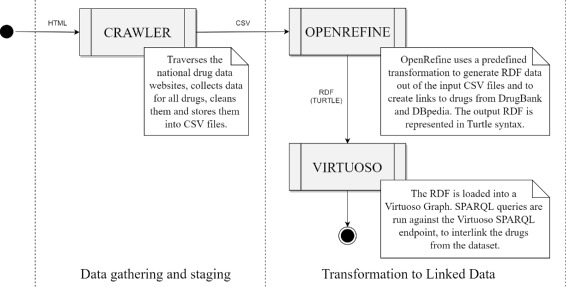
Workflow: Transforming 2-star data from different national drug data registries to 5-star Linked Drug Data

The automated system gathers the data, performs data cleaning, aligns the data with the predefined schema skeleton, uses the transformation script and the SPARQL query to transform the data to RDF, extend it with missing ATC codes and add links to drugs from the same dataset and drugs from DrugBank and DBpedia, thus turning the dataset into a Linked Data dataset. The workflow of these actions is depicted in Fig. 3. These steps represent the activities defined in Step III of the methodological guidelines.

**Data gathering and staging**. In order to create a sustainable system for Linked Drug Data, we had to design a way to collect the necessary data from the national drug registries, on a scheduled basis. Therefore, we developed a set of specialized web crawler applications which crawl the designated drug registry websites, collect the necessary data, clean it and store it in a predefined CSV format (Fig. 3). We need to use a set of such crawlers as the target websites differ in structure and available data. The output CSV files from the crawlers use the predefined CSV structure described above.

Like most of the data available on the Web, the drug data available on the national drug registry websites is not evenly structured, nor completely clean. We therefore needed to extend our crawlers with functionalities which perform data cleaning tasks and work to detect data from all variations of the source webpages.

As per the suggestions in Step III of the guidelines, we used their existing webpage URLs of the drugs as their unique URIs, e.g. https://lekovi.zdravstvo.gov.mk/drugsregister/detailview/53457. As many of the drug entities contain information about more than one generic name, manufacturer, active ingredient and strength, the crawlers are tasked with splitting them into the corresponding columns in the CSV files. Additionally, information such about the cost of the drug and its strength are split into separate fields, to match the CSV template. The crawlers also take care about the specific formats needed for some of the columns, such as the dates, the country codes, the currency codes, and the prescription status.

The drug data from the several of the countries were an exception, as they are available for download as Microsoft Excel or PDF files. For these datasets, the crawlers have to restructure the columns from the source data and generate a CSV file with the correct column names according to the CSV template. For these drugs, we generate custom URIs as identifiers, which have the format http://linkeddata.finki.ukim.mk/lod/data/loddw/drugs/countryCode#drugID. Here, drugID is an ID generated by the crawler, countryCode is a three-letter country code (according to [63]) of the country where the drug was registered and the other parts of the URI identify the project and the datatype on our Linked Data website: /lod/data/loddw is the project and /drugs is the categorization of the data.

The result of this stage in the workflow, in our case with the twenty-three selected countries, is a set of twenty-three separate CSV files which follow the CSV template. The only difference is that some of the CSV files can have no values in some columns, due to the data being unavailable online. When we get all twenty-three CSV files, the first part of the workflow is done and we can continue with the second part.

**Transformation to Linked Data**. The CSV files can be combined into one CSV file, or remain separate. The only difference will be in the performance of the next step which can be done as a single longer process, or as twenty-three separate and shorter processes. To keep the processing time per transformation shorter, we use twenty-three separate CSV files, each representing the drug dataset of a different country.

We send the twenty-three CSV files to a BatchRefine instance [64], which represents a wrapper over an OpenRefine instance with the RDF extension, that can be used as a REST-based service. The HTTP POST calls to the BatchRefine REST-based service are done with the reusable BASH script developed as part of the methodology supporting tools and contain (a) the CSV file which needs to be transformed and (b) the OpenRefine transformation script defined as a supporting tool for the guidelines. The result of the call is a transformed RDF output, which contains part of the generated Linked Drug Data dataset.

The output of our BatchRefine transformations are twenty-three RDF files in Turtle format. These RDF files are a Linked Data dataset: they contain 5-star data about the drugs from the twenty-three countries, along with links to generic drugs from the LOD Cloud - more specifically, links to generic drugs from both DBpedia and DrugBank. As we will see later in the text, we can use these links to fetch data about the drugs from our dataset which we don’t have on our end and which do not exist on the source national drug registry websites, but can be found in other datasets on the Web and can potentially prove to be of interest for the end-users.

After the transformation with BatchRefine is done, we load the RDF files into a Virtuoso instance [61] using a BASH script. All RDF files are loaded into the same RDF graph. The latest run of the workflow (Fig. 3) resulted in 248,746 distinct drugs in this step, represented with a total of 7,245,294 RDF triples and with 278,542 outgoing links to drugs from the LOD Cloud.

After the RDF data has been stored into an RDF graph in Virtuoso, we use the SPARQL queries for extending the dataset with missing ATC codes and interlinking related drugs. We execute the SPARQL queries over our dataset stored in Virtuoso, using a BASH script. In the latest run of the workflow (Fig. 3), 38,758 new ATC codes were added for drug products which did not have an ATC code in the source registry. Then, 91,782,500 schema:relatedDrug triples were added between similar drug products, i.e. 45,891,250 pairs of drugs from our dataset were identified to have the same function, but exist under different brand names, are from different countries, or are produced by different manufacturers, or maybe have a different packaging size, strength, cost, etc. As we will see further in the text, we can utilize these interlinkings for providing the users with alternative drugs they may aquire for treating their condition, either in the same of in a different country.

The workflow shown in Fig. 3 is activated on a scheduled period, currently set at one month. In order to handle the data changes during updates, we backup the RDF graph holding our dataset and replace it with the newly created RDF graph. With this we employ versioning and maintain the default graph identifier to always denote the latest version of the LinkedDrugs dataset.

According to the recommendations in Step IV of our guidelines, the dataset needs to be published on the Web, where it will be publicly available. Therefore, we published our LinkedDrugs dataset according to the best practices for publishing Linked Data [50], via a permanent, dereferenceable URI, which supports HTTP content negotiation: http://linkeddata.finki.ukim.mk/lod/data/drugs [65]. The dataset is hosted at a live Virtuoso instance [61], in a named RDF graph <http://linkeddata.finki.ukim.mk/lod/data/drugs#> which holds the latest version of the dataset, publicly available via a SPARQL endpoint [62] which serves as a REST-based service. Additionally, data dumps of the dataset are available on Datahub [55].

#### Usage scenarios

With the automated system and its workflow we start with twenty-three different, distributed and browsable datasets, available on the Web and intended for human consumption via HTML webpages, and using the methodological guidelines and tools we manage to create a consolidated dataset of interlinked and schema-aligned drug products from different countries, additionally linked to generic drugs and active ingredients from the LOD Cloud. In order to demonstrate the advantages of having the drug data in a 5-star data quality format, and therefore implement the recommendations from Step V of the methodological guidelines, we will show a few use-case scenarios via SPARQL queries. The most basic scenario would be to select all data about a single drug of interest, which is very simple and straight-forward, and therefore omitted here. Since the Virtuoso SPARQL endpoint at [62] can be used as a REST-based service, these SPARQL queries could be used from any type of application to always access and exploit the latest data available.

**Interlinked drug data**. The interlinking created between the drugs from the dataset, with the schema:relatedDrug triples, can be utilized in a use-case which allows the end-users to discover drugs from the same country or another country which have the same therapeutic, pharmacological and chemical properties as the drug of his/her interest. This is a useful feature when the drug of interest is not available or when the user is traveling abroad. Getting information about drugs with the same properties and their respective prices can be useful for determining the drug from a specific category which is affordable in the specific case. This can be used by pharmacists, doctors and even patients for gathering information and determining the appropriate treatment.

An example SPARQL query which can be used to identify the drugs from all countries which have the same therapeutic, pharmacological and chemical properties as the drug of interest, is shown below (Query 1). 

In the query, only a handful of data of interest of the related drugs are selected, but depending on the specific use-case, they can be expanded. Query 1 can also be modified to include the specific drug of interest, by modifying the drug URI in line 8. In our example in Query 1, we use the Norwegan drug “Airomir” as an example, and get results for over 300 distinct and related drugs from many different countries in the dataset. The query and its full results can be viewed online on Seminant [66], at http://seminant.com/queries/5803e77573656d19eb6c5d00. Partial results are shown in Table 1.

**Table 1:** Partial results from Query 1

Drug product	Manufacturer	Country
Activent Sr	Medical Union Pharmaceuticals - Egypt	EG
Aerolin 100mcg/dose Inhaler		EG
Aeroline 400 Inhaler		EG
Aerotropa	Pharco B International-egpyt	EG
Agolin	Agog Pharma Ltd	UG
Airomir	iNova Pharmaceuticals (New Zealand)	NZ
Airomir Autohaler	Teva Sweden AB	NO
Airomir Autohaler	iNova Pharmaceuticals (New Zealand)	NZ
Airomir Autohaler 100 microgramos	Teva Pharma S.L.U.	ES

**Linked LOD drug data**. The main advantage of having links between data from different and distributed datasets is the ability to query them from a single point, over the existing infrastructure of the Web, using W3C standards such as HTTP, SPARQL and RDF. As we have rdfs:seeAlso links from drugs in our dataset to corresponding generic drugs from the DrugBank and DBpedia datasets, we can utilize them to get additional information about the drugs from our dataset whenever we are browsing them. Such additional information will come from the DrugBank and DBpedia datasets, and can include additional drug description, the interactions the drug has with other drugs or with certain foods, the drug mechanism of action, the drug pharmacology, absorption, biotransformation and toxicity, the list of alternative brand names and a list of webpages for the drug on other locations on the Web. This data is not available on the original national drug registry websites, which are the source for our dataset; it is data retrieved directly from the distributed DrugBank and DBpedia dataset, using SPARQL federation [67].

An example of a federated SPARQL query which selects information about a drug of interest from the DrugBank and DBpedia datasets is shown below (Query 2).



The query selects some very important data about the drug of interest and its active ingredient from DrugBank and DBpedia. The most significant are the chemical, biological and pharmacological properties of the drug, along with its interactions with food and with other drugs. This data is not always available on the national drug data registries, but is of high importance for the end-users, especially the pharmacists and doctors who may require them when determining treatment for acute conditions of a patient who is already on a treatment of a chronic medical condition.

An example run of Query 2, for the drug product “Duloxetina” from Spain, results in details for the generic drug “Duloxetine” from both DrugBank and DBpedia: http://seminant.com/queries/5803e9b973656d19eba65e00. Among other details, it also shows the 3 specific food - drug interactions the drug is involved in, along with the 13 drug - drug interactions it has. Partial results from the query are shown in Table 2.

**Table 2:** Partial results from Query 2

Description (from DBpedia)
Duloxetine (Cymbalta, and generics) is a serotonin-norepinephrine reuptake inhibitor (SNRI) created by Eli Lilly. It is mostly prescribed for major depressive disorder, generalized anxiety disorder, fibromyalgia and neuropathic pain. Duloxetine failed to receive US approval for stress urinary incontinence amid concerns over liver toxicity and suicidal events; however, it was approved for this indication in the UK, where it is recommended as an add-on medication in stress urinary incontinence instead of surgery.
Food Interactions
Food does not affect maximum levels reached, but delays it (from 6 to 10 hours) and total product exposure appears to be reduced by only 10 percent. People taking this product who drink large amounts of alcohol are exposed to a higher risk of liver toxicity. Take without regard to meals.
Drug Interactions
Amitriptyline: Possible increase in the levels of this agent when used with duloxetine.
Ciprofloxacin: Ciprofloxacin increases the effect/toxicity of duloxetine.
Desipramine: Possible increase in the levels of this agent when used with duloxetine.
Flecainide: Possible increase in the levels of this agent when used with duloxetine.
Fluvoxamine: Fluvoxamine increases the effect and toxicity of duloxetine.
Imipramine: Possible increase in the levels of this agent when used with duloxetine.
Isocarboxazid: Possible severe adverse reaction with this combination.
Nortriptyline: Possible increase in the levels of this agent when used with duloxetine.
Phenelzine: Possible severe adverse reaction with this combination.
Propafenone: Possible increase in the levels of this agent when used with duloxetine.
Rasagiline: Possible severe adverse reaction with this combination.
Thioridazine: Increased risk of cardiotoxicity and arrhythmias.
Tranylcypromine: Possible severe adverse reaction with this combination

**Analytics**. Aside from the use-case scenarios for end-users, our LinkedDrugs dataset can be used for analytical queries as well. These analytical queries allow interested parties to gain insight into the drug markets of different countries, allowing them to analyze the available consolidated data using a single entry point for querying and using a single query language. The analytics could be built-in in specific analytic applications, or can be executed with separate and standalone SPARQL queries.

To get a better understanding of the analytical possibilities over consolidated drug data from multiple countries, we will look at an example query which identifies the most common drug categories per country. This would allow the user, e.g. pharmaceutical company, to gain a better knowledge on the national drug markets and make an informed decision about placing their drug in the country of interest. A general SPARQL query for this analytical scenario is given below (Query 3):



A sample run of Query 3 shows that Romania, Spain, Netherlands, Ireland and Slovakia have most drugs (5,362, 2,152, 1,488, 758 and 709, respectively) in the category of agents acting on the renin-angiotensin system (ATC C09), Russia and South African Republic have most drugs (1,536 and 976, respectively) in the category of antibacterials for systemic use (ATC J01), while USA has 1,270 drugs in the psycholeptics category (ATC N05). These partial results are shown in Table 3. The full results from the query are available at http://seminant.com/queries/5803ebc473656d19ebac5e00.

**Table 3:** Partial results from Query 3

Drugs	ATC Prefix	Country
5362	C09	RO
2152	C09	ES
1536	J01	RU
1488	C09	NL
1270	N05	US
976	J01	ZA
758	C09	IE
709	C09	SK
707	N02	NZ

Another analytical scenario would be to assess the average drug price per drug category, per country. It could be used by medical authorities in a country to determine the cost situation per category in other countries and use the information for local regulations. It could also be used by a pharmaceutical company to determine the price range for a new drug, before it goes to market. An example SPARQL query which can be used for such an analysis is given below (Query 4):



A sample run of Query 4 identifies that the ATC drug categories with the highest average price in Norway are drugs for disorders of the musculo-skeletal system (ATC M09), respiratory system products (ATC R07) and alimentary tract and metabolism products (ATC A16). In Macedonia they are alimentary tract and metabolism products (ATC A16), pituitary and hypothalamic hormones and analogues (ATC H01) and antihemorrhagics (ATC B02), while in Australia and Slovenia they are respiratory system products (ATC R07) and in South African Republic they are immunosuppressants (ATC L04), hematological agents (ATC B06) and alimentary tract and metabolism products (ATC A16). These partial results are shown in Table 4, while the full results which include other countries as well, are available at http://seminant.com/queries/5803ed6e73656d19eb537e00.

**Table 4:** Partial results from Query 4

Avg. price	Currency	ATC Prefix	Country
93480.60	NOK	M09	NO
47221.40	NOK	R07	NO
39557.30	NOK	A16	NO
32021.40	MKD	A16	MK
28837.20	MKD	H01	MK
27478.20	MKD	B02	MK
22500.00	AUD	R07	AU
17822.00	SVN	R07	SI
13360.10	EUR	V10	CY
10679.50	ZAR	LO4	ZA
10127.10	ZAR	B06	ZA
9880.81	ZAR	A16	ZA

In cases when an inter-country comparison of the pricing is necessary, an application could use a currency converter to transform the values to the same currency of choice, and make the comparison.

## Discussion

The generated LinkedDrugs dataset aims to be the first comprehensive, consolidated and aligned dataset of drug product data on a global scale. In its first version, the dataset consisted of only seven countries; currently, that number has grown to twenty-three. As we have the methodology and the tools in place, the addition of new countries is more or less straightforward. Even though Linked Data datasets with drug data already exist in the LOD Cloud, they consist solely of generic drugs, i.e. active ingredients. Contrary to this, our LinkedDrugs dataset consists of drug products which are registered in a specific country, have a specific name, dosage form, amount, strength, barcode, manufacturer, license, price, ATC code, etc. Since these drug products from our dataset are linked to the existing generic drugs and active ingredients from the LOD Cloud, the novel usage scenarios greatly exceed what is currently possible with Linked Drug Data on the Web.

The developed methodological guidelines and supporting tools intend to encourage and lower the boundaries for data publishers from the domain to contribute to the LinkedDrugs dataset, further extending its potential and value. Since the adoption of the developed tools can present a potential hurdle, we believe that the transformation process presented in this paper can serve as an additional guide.

Our methodological guidelines extend the existing Linked Data methodologies, briefly presented in the section “Background”. Here, we will make an explicit comparison of the similarities and differences in the approaches among the existing methodologies, and our findings and proposal to group them into five general steps. These five general steps have allowed us to define our own methodological guidelines for the drug data domain, by defining tasks and tools specific for the domain and adding them to the corresponding general steps. These specific tasks and tools have been the result of our research in the domain, as well as our previous work with generating, publishing and using healthcare Linked Data datasets.

The existing Linked Data methodologies have a varying number of steps, but still generally cover the same activities. The main difference in the methodologies is the grouping of actions within different steps and on different levels of granularity. Apart from some explicit differences, which we will further examine, they cover the palette of actions involved in the process of generating and publishing a Linked Data dataset, and thus can be grouped into five general steps.

From Table 5, we can see that all existing Linked Data methodologies define the knowledge of the data, its domain and the existing datasets from the LOD Cloud as the first step(s). The actions they cover in the steps relate to these tasks, and while some methodologies focus on the data domain, others recommend knowledge of existing Linked Data datasets, as well.



The second step refers to the tasks of modeling the data, locating and selecting the appropriate ontology or vocabulary, extending existing ontologies and vocabularies to match the original data schema, creating a custom ontology or vocabulary from scratch and mapping it to existing ontologies, and defining the URI naming scheme for the ontology classes and properties. The methodologies of Hyland et al. [51], Hausenblas et al. [52] and Villazón-Terrazas et al. [53] explicitly define these tasks, whereas the LOD2 methodology does not define them. For this step, we defined an RDF schema which can be reused when working with drug data from national drug registries or other sources. The provided CSV template allows the data publisher to align the data in the correct format in a CSV file, and prepare it for the next step.

The third step is focused on transforming the source data into RDF, creating links and 5-star Linked Data datasets, and creating metadata descriptions of the dataset and its links to other datasets. All four methodologies define these tasks, with the LOD2 methodology being the most specific one. The LOD2 methodology, being the latest one, understandably contains activities which include classification, quality control, data evolution and versioning. The use of the VoID vocabulary is explicitly stated in this phase in the methodologies of Hyland et al. and Hausenblas et al. The methodology of Villazón-Terrazas et al. defines this task in its next step, ‘4. Publish’, but as it contains other tasks which better fit in our next step, we left it out of this one. Here, we developed an OpenRefine transformation script which can be used with any source drug data formatted with the CSV template from the previous step, in order to get high quality, 5-star Linked Drug Data. For the purpose of generating additional links between similar drugs in the dataset itself, we developed a SPARQL-based tool which can be used over any Linked Drug Dataset generated with the OpenRefine transformation script.

The fourth step defines the tasks for publishing the dataset on the Web. The exact actions which should be undertaken are defined in the methodologies of Hyland et al. and Villazón-Terrazas et al. The other two methodologies do not define such tasks and steps. In order to simplify this step for data publishers, we provide a web-based tool for automated publishing of the generated Linked Drug Data dataset. The tool also interlinks the new dataset with the consolidated LinkedDrugs dataset, which already contains drug product data from twenty-three national drug repositories.

The fifth step consists of tasks for defining use-case scenarios or development of specific applications and services which take advantage of the newly created Linked Data dataset and its links to other datasets. The methodology of Hyland et al. does not specify such an activity, arguing that the data publisher does not have to think about potential use and reuse of the data while working on generating Linked Data from the source dataset. According to them, the data publisher should just make sure he/she correctly transforms, describes and publishes the data, and let the users and the interested community know about the dataset. However, Hausenblas et al., Villazón-Terrazas et al. and the LOD2 Project team explicitly state that the last step of the Linked Data generation process should consist of defining use-cases and/or developing applications and services.

The tools we developed as support for the steps of the methodology aim to help data publishers from the domain. They can aid both data publishers from the drug domain which do not necessarily have prolific experience with Linked Data, as well as Linked Data publishers which are not very familiar with the domain of drugs and healthcare. These open-source and reusable tools are supposed to lower the bounds for interested parties to get involved in the domain and include their datasets in the global LODD and LOD Cloud.

## Conclusions

The amounts of data available on the Web represent a goldmine for data-driven applications and services [68]. Unfortunately, the data is available in different formats and is distributed over various locations. This is a huge obstacle which blocks progress in data retrieval in many domains. The healthcare domain is no different: the national drug authorities publish their data on the Web in different formats and granularity levels, and there is no comprehensive method for retrieving and using them.

The Linked Data concept provides new ways of publishing and connecting data from different distributed sources, which allows data consolidation. It also provides a new spectrum of use-case scenarios which can be useful for generating new business value for businesses and independent developers, by allowing them to develop innovative applications and services in the domain. The opportunities which lie within the creation of new use-case scenarios from Open Data and Linked Data are a field whose potential is becoming increasingly recognized [69].

Motivated by this, we propose methodological guidelines for using Linked Data principles to consolidate drug data, and provide a set of tools which intend to aid the data publishers in the steps of the methodology. Our methodological guidelines extend the existing Linked Data methodologies, both general and aimed for government data. We combine the steps from the existing methodologies into five steps which are necessary for developing a sustainable Linked Data dataset. Each of these steps is extended to include specific tasks, actions and tools which are important for the drug data domain. The aim of our methodological guidelines is to enable the creation of Linked Drug Data datasets which are very well aligned between themselves and which can then easily be consolidated into a comprehensive system or dataspace.

We implemented our guidelines and tools as part of an automated system for transformation and publishing of the 2-star and 3-star data from twenty-three national drug registries into consolidated and aligned 5-star Linked Drug Data. The system provides regular updates which make sure the created LinkedDrugs dataset always contains new data. In order to create the dataset, we use the defined common RDF schema, the CSV template, the OpenRefine transformation script, as well as the SPARQL queries for extending the dataset and interlinking the drugs from the dataset. With this, we ensure data alignment with other LOD and LODD datasets. Currently, our generated LinkedDrugs dataset contains over 99,000,000 RDF triples, which represent data for over 248,000 drug products from twenty-three countries. The dataset also contains over 91,000,000 schema:relatedDrug relations between the drugs, and over 278,000 rdfs:seeAlso relations to generic drugs and active ingredients from DrugBank and DBpedia.

We further present new usage scenarios enabled by Linked Data, aimed both for end-users and analytical purposes, which utilize the consolidated and aligned nature of the dataset and its contextual links to entities from the LOD Cloud. We achieve this by automatically generating the additional relations in our dataset which link the drugs between themselves, and link the drugs with drug entities published as part of other LOD Cloud datasets. Using W3C standards and the Semantic Web technologies, we are then able to retrive data from these distributed datasets, and present them to the end-users in a comprehensive manner.

As future work, we plan to extend the number of national drug registries from the current twenty-three. The design of the supporting system requires that we only modify the data gathering and staging part of the workflow, while the transformation and publishing process remains the same. We also intend to extend the reconciliation tasks in OpenRefine, by employing reconciliation for the company names. This would provide additional links in the dataset between the drugs and the manufacturers, represented by company entities on the LOD Cloud. Given that the names of the companies and their details are sometimes on local languages, depending on the drug registry, we would also add a translation service in the workflow. We also plan to add an automated VoID metadata generation task for the dataset, which is a recommendation from Step III. Regarding applications built on top of the datasets, we have already started developing user-focused and analytical applications which provide the end-users with detailed information about a drug product and its interaction with other drugs and foods, but also provide insight into the drug product availability on a global scale. All of these additional features would further add to the benefit of having consolidated and aligned drug data from various countries in one place, accessible via the existing infrastructure of the Web and via existing W3C standards.

## Methods

### The methodological guidelines

Based on our experience with applying the Linked Data principles in the healthcare and drug domain and on the existing Linked Data methodologies, we developed a set of methodological guidelines for consolidating drug data using the Linked Data approach. These guidelines improve upon the existing Linked Data methodologies and contain steps, activities and tools which are specific to the drug data domain. Their purpose is to guide data publishers through the process of generating high quality, 5-star Linked Data in order to interlink, align and consolidate drug data from different national drug registries or other sources of drug data. The alignment and relationship between the existing methodologies and our guidelines is outlined in Table 5.

Along with the guidelines, we have developed a set of tools which simplify the execution of the specific steps of the methodology. Their intention is to support the Linked Drug Data generation process for both people from the drug domain which do not have deeper knowledge of Linked Data, and Linked Data publishers which do not have deeper knowledge of the drug domain. These tools are open and publicly available on GitHub [58].

Our methodological guidelines consist of the following steps (Fig. 1):

I. Domain and Data Knowledge
II. Data Modeling and Alignment
III. Transformation into 5-star Linked Data
IV. Publishing the Linked Data dataset on the Web
V. Use-cases, Applications and Services

These steps have been developed with reuse as a primary goal; therefore, their main focus is the encouragement of data publishers in the drug domain to develop, modify and use reusable components during the steps of the methodology. This makes the Linked Drug Data lifecycle modular, i.e. constructed of loosely-coupled components which can be reused in the domain. These loosely-coupled components can be used separately when necessary, but also form a seamless workflow for generating a high-quality, 5-star Linked Drug Data dataset. The reuse of such components, like in other software development cases, reduces development time and increases productivity [56, 57].

#### Step I: Domain and data knowledge

The first step corresponds to the first steps from the existing methodologies: it is important for the data publisher to know the domain and the data in it very well. This understanding of the data source schema and semantics is crucial for the following steps which will involve data modeling, schema alignment and data transformation.

In the drug data domain, if this is the first time the data publisher comes across Linked Data, our advice is to first get familiar with the 5-star data system from Tim Berners-Lee [35], the four principles of Linked Data [70], and the LOD Cloud [32]. After that, a study of the LODD Project [36], the Bio2RDF Project [43] and the DrugBank Linked Data dataset [44] is recommended. This will help the data publisher to get a better insight into the Linked Drug Data and Linked Healthcare Data domains, the types of data which exist in them, their schema, their similarities and differences and their existing and potential links. It will also help him/her determine the ontologies and vocabularies already used in the domain, which can be important for the next step.

In a general case, when working with any other domain, it is important for the data publisher to get familiar with the domain in question and with the meaning of the dataset selected for transformation. For this, a consult with a domain expert is usually necessary and therefore advised. Another approach is to explore the existing Linked Data datasets which are similar to or from the same domain as the one of interest. For this, the Datahub portal [71] and the LOD Cloud cache instance [72] could be used.

#### Step II: Data modeling and alignment

In the next step, the data publisher should focus on data modeling and alignment with other existing or future datasets. The data publisher has to choose the correct schema for the dataset, in order to annotate it correctly, i.e. use the data fields which are necessary for the final use-cases, annotate the fields unambiguously and with the correct semantics and make the correct schema choices which will allow seamless alignment with other datasets. Additionally, the data publisher has to define the URI naming scheme for the data entities, and optionally for the ontology or vocabulary classes and properties.

**Data schema**. In the drug data domain, studying the datasets from the LODD and Bio2RDF projects can help get an insight into the ontologies and vocabularies used in the domain. Some of the ontologies and vocabularies which a data publisher needs to have in mind are: Schema.org [59], DBpedia Ontology [73], UMBEL [74], DICOM [75], the DrugBank Ontology used for the data at [44], as well as other biomedical ontologies [76].

In order to support the data publishers from the drug domain in this step, we designed a reusable RDF schema for the data, shown in Fig. 2. The schema can be used by data publishers working with drug data from national drug registries or other sources. The schema is comprised of classes and properties from the Schema.org vocabulary [59]: the schema:Drug class, along with a large set of properties which instances of the class can have, such as name, code, activeIngredient, nonProprietaryName, availableStrength, cost, manufacturer, relatedDrug, description, url, etc. Additionally, in order to align the drug data with generic drugs from DrugBank, we use the properties brandName, genericName, atcCode and dosageForm from the DrugBank Ontology. In order to annotate the links which the drug product entities will have to generic drug entities from the LOD Cloud dataset, the rdfs:seeAlso relation is used.

In general, when working with data from another domain, the data schema is defined with the choice of vocabularies or ontologies to be used. The principles of ontology engineering and usage have been developed for this purpose exactly: to maximise the chances of reuse, and therefore allow better alignment between datasets [77]. This means the agent should always try to reuse an existing vocabulary or ontology, giving advantage to those which are most widely used. A few tools for ontology and vocabulary discovery exist, and the data publisher should use them in this stage. The two most notable are Linked Open Vocabularies (LOV) [78] and DERI Vocabularies [79], which also provide usage statistics which can be used to assess the impact of a given vocabulary or ontology in a specific domain. Our choice of the Schema.org vocabulary follows the reusability paradigm: it is the most widely and generally used vocabulary across the Web.

However, datasets tend to have specific fields, which are not covered by existing ontologies. In this cases, the existing ontology or vocabulary should be extended, or a new one should be defined. However, each time a new ontology is developed, it is important to define the mappings between the new classes and properties and the classes and properties from other ontologies, in order to enable ontology matching and RDF-based reasoning, for schema alignment. In order to avoid defining specific new properties, we reused some properties from the Schema.org vocabulary which are currently not explicitly intended for use with schema:Drug entities. An example of such properties is the schema:addressCountry property which should be used for an address, but we use it in our schema to denote the country in which the drug is registered.

Another important approach in this step is the use of upper-level ontologies and vocabularies; they can provide a schema for many different and specific domains, due to their generality. Having two or more datasets annotated with the same upper-level ontology or vocabulary allows interlinking and inference between them, i.e. it improves the alignment which is crucial for data consolidation.

**Data alignment**. For the data alignment task, the data publisher needs to make sure that the generated dataset will be well aligned with existing and future datasets from the same domain. In order to aid the data publishers with this, as well as help them in preparing the drug data for the transformation step, we developed a CSV template intended for the drug domain [58]. This CSV template can be used with the drug data and is comprised of the fields necessary for applying the RDF schema from Fig. 2.

The data publisher interested in publishing Linked Drug Data should use this CSV template for the data, following the specifics defined for each of the fields. These specifics assure that the data will be of high quality and completely aligned with other drug data generated using the same methodological guidelines.

**URI formats**. From the URI naming scheme perspective, in the domain of drug data it is important to determine the types of entities which exist in the dataset. This will help in defining the entity URIs for the Linked Data dataset. According to the Linked Data principles, each entity in the dataset - along with the classes and properties in the ontology - needs to have a unique indentifier in the form of an HTTP URI. In order to provide better performance when using the dataset in the future, our experience suggests using separate URL paths for different entity types, e.g. http://example.com/drug/, http://example.com/interaction/, http://example.com/disease/, etc. An additional recommendation is to use slash-based URIs, instead of hash-based ones. This may result in using an additional HTTP request by the machine accessing the URI, but it provides better performance when accessing large datasets [80].

However, to simplify this step for the drug data publishers, we advise the use of the existing webpage URLs of the drugs from the national registry websites, which are already unique. According to the Linked Data principles, the entity URI should denote a Web location where the end-users and agents can get more information about the entity, so our approach satisfies the condition.

#### Step III: Transformation into 5-star Linked Data

During the third step, the source dataset should be transformed into a 5-star Linked Data dataset. The process of transformation can be executed in many different ways, and with various software tools, e.g. OpenRefine [81], LODRefine [82], D2R Server [83], Virtuoso [84], Silk Framework [85], etc.

To help the data publishers from the drug domain and to automate this step, we developed a reusable OpenRefine transformation script [58]. This transformation script is specifically designed for the drug data domain, and the RDF schema and CSV template from Step II. It contains a set of actions which generate RDF from the inputed CSV file which contains drug data. In the process, it also locates associated generic drugs from the DrugBank and DBpedia datasets for each drug product in source dataset, and extends the generated RDF with links between the drugs from the dataset and the corresponding drugs from the LOD Cloud.

The transformation script can be reused with any OpenRefine instance which has the RDF extension. It can be applied on any drug data dataset formatted with the CSV template from Step II. As a result, it will generate a Linked Drug Data dataset annotated with the RDF schema from Step II (Fig. 2).

The RDF schema from Step II defines relations between the drug products from the dataset as well. These relations are denoted with the schema:relatedDrug relation (Fig. 2). In order to provide means for generating RDF triples which interconnect the drugs from the dataset, we developed a SPARQL query [58] which can be executed over the dataset generated with the OpenRefine transformation script. The SPARQL query detects all pairs of drugs from the dataset which have the same ATC code - and therefore have the same therapeutic, pharmacological and chemical properties - and creates two triples connecting the first drug to the second one with the schema:relatedDrug property, and vice-versa.

In a general case, in order to make the correct choices about the tools to be used for the transformation process, it is important to distinguish the characteristics of the dataset first. The nature of the dataset will determine if (a) the transformation is a one-time task, a task which will have to be executed on a given time interval (e.g. once a month), or a continually running task; (b) old versions of the transformed dataset are necessary for versioning and as backup, if during future transformations only the changes in the data are needed for transformation, i.e. ‘delta’ updates are performed, or if older data are no longer necessary for the particular use-case; (c) manual or automated data cleansing is needed before the first transformation and/or subsequent transformations; (d) the source dataset is always available at the same location and is accessible via the same interfaces. These specifics of the dataset in question can then help the data publisher determine if the transformation task can be fully or partially automated, and identify the parts of the transformation workflow which require human attention and input.

Adding metadata about the newly created Linked Data dataset is significant from the data reuse perspective - using vocabularies such as VoID [86] help ubiquitously determine the characteristics of the dataset and the links the dataset has to other Linked Data datasets, through software agents. VoID metadata contains information about the name, description and category of the dataset, versioning information and update frequency, contact information, the license under which the dataset is made available, the links to the SPARQL endpoints and URI lookup endpoints, used vocabularies and their properties and classes. It also explicitly defines the links between the dataset and other Linked Data sets, defined in the dataset itself. The use of the VoID vocabulary is explicitly stated in the corresponding steps in the methodologies of Hyland et al., Hausenblas et al. and Villazón-Terrazas et al.

In the domain of drug data providing new and updated data is very important - old data has no importance to the end-user, except for analytics. This means that the data publisher should anticipate the change rate of the source dataset and correctly design the workflow of refreshing data from the source dataset to the Linked Data dataset. This would translate to creating a sustainability plan which will transform new data and add it to the Linked Data dataset, remove old data and provide versioning. Depending on the size of the source dataset, the data publisher can choose to re-transform the source dataset on each update, or to provide means for performing ‘delta’ updates. Providing versioning is also important, as new transformations can sometimes result in errors, rendering the dataset unusable.

#### Step IV: Publishing the Linked Data dataset on the Web

In the forth step, the generated 5-star Linked Data dataset, along with its VoID metadata, should be published on the Web. This should be done following the W3C recommendations for publishing Linked Data on the Web [50], which suggest enabling direct URI resolution, providing a RESTful API, providing a SPARQL endpoint, and/or providing the dataset as a file for download.

There is a large palette of tools and software platforms which allow seamless Linked Data publishing. Among them are D2R Server [83] and Virtuoso [84], which allow Linked Data publishing of datasets which are originally in an RDF file (Turtle, N3, RDF/XML, JSON-LD, etc.), a CSV file, or in a relational database. These platforms then allow access to the Linked Data dataset via HTML pages, via RDF file downloads and via a SPARQL endpoint which can be used as a RESTful API as well.

When creating a Linked Drug Data dataset, we recommend adding and interlinking it with the global LinkedDrugs dataset which will consist of all such datasets generated by different parties, using these guidelines. To enable this, we have developed a web-based tool for uploading the generated datasets [60], which after a human-based quality assessment triggers an automated process for interlinking the new dataset with the existing LinkedDrugs datasets and publishing it according to the Linked Data principles and best practices.

We also recommend publishing the dataset at Datahub.io [71] under the healthcare and drugs categories, as well as adding the #linkeddrugs tag. Additionally, we advise joining the LOD Cloud [87]. Both these actions will enable higher visibility of the dataset.

Another important part of this step is the announcement of the newly created Linked Data dataset to the public. For this, information about the dataset along with its VoID metadata should be published on popular data portals, such as Datahub.io [71]. This announcement should also be done via existing communication channels of the data publisher and his/her organization. In order to facilitate further use and reuse of the dataset, it is important to provide a form or a contact email address for interested parties to be able to report data or access issues, and provide feedback. On the organization side, it is important that these reports and requests are attended to in a timely fashion; otherwise the usability of the dataset is significantly lowered.

#### Step V: Use-cases, applications and services

The last step refers to defining use-case scenarios and/or developing specific applications and services which will use the data from the newly created Linked Data dataset, to showcase the (re)usability of the dataset and its links to other Linked Data datasets. This will present the potential of the contextually linked datasets to future interested parties.

When creating a Linked Drug Data dataset, potential use-case scenarios, applications and services should include contextually linked data from the LODD datasets and the Bio2RDF datasets. The LODD datasets, especially the DrugBank linked dataset, contain data about generic drugs, i.e. active ingredients, along with their pharmaceutical and pharmacological properties, targets, brand names, food interactions, drug interactions, etc. Since the national drug data registries contain information about drug products, one-to-one mappings between the entities from such datasets and the DrugBank and DBpedia datasets are not possible. Instead, using our RDF schema from Step II and the OpenRefine transformation script from Step III, each entity from the dataset will be linked to one or more generic drugs/active ingredients from DrugBank and DBpedia, based on its ATC code [88]. This way, the drugs from the dataset get a contextual link to the generic drugs, and from there, to all of its properties and characteristics. Additionally, the existing links from the DrugBank and DBpedia generic drugs to other healthcare datasets can be further exploited, as they also represent contextual links. Such links currently point to LinkedCT and Bio2RDF. To demonstrate the usability of the generated Linked Drug Data datasets, we provide example use-cases on the project website [60] and on GitHub [58].

In a general case, the use-cases can be text-based scenarios, specific SPARQL queries, or prototype applications, describing the ways in which the data from the new dataset can be browsed, retrieved and used. Here, a specific focus should be given on how the links to other Linked Data datasets can be exploited to reach other data, not present in the original data source, to extend its context. With this, the data publisher will show to interested parties that the original dataset has more value when combined with datasets from the same or similar context, instead of being used in an isolated scenario. Besides such use-case, the same effects of the Linked Data dataset can be showcased with the development of applications and services. They bring more visibility to the general (re)usability of the Linked Data dataset, but generally require more time and effort.

The created use-cases, applications and/or services, should be shared and announced to the public, along with the dataset itself and its VoID metadata. The use of the same channels from the previous step is advised.

### Methodology supporting tools

As part of the methodological guidelines, with the intent to provide help to the data publishers working in the drug data domain, we designed and developed a set of tools. These tools consist of the RDF schema, the CSV template, the OpenRefine transformation script, the SPARQL-based tool for interlinking related drugs and the web-based tool for automated transformation, interlinking and publishing of the generated Linked Drug Data dataset.

#### RDF schema

In order to model the domain of drug products on a global scale, we needed to create one common schema for all national drug data repositories, and then use it for annotating the drug data. With it, the goal was to provide alignment of drug data from different sources, with different format and different levels of data granularity, in order to enable simpler data exploitation.

First, we analyzed the national drug data repositories of 31 countries^1^ and the analysis helped us define a common set of properties which exist and which we want to use in our dataset. This set consisted of 24 properties, including the brand name of the drug, the generic name, the ATC code, the EAN code (barcode), the list of active ingredients, the drug strength, dosage form, cost, manufacturer, the country it was registered in, the details about its license, etc. Not all national drug data repositories provide all of the data and properties we selected for our schema, but we did not want to decide against using those properties - they are useful where available.

Following the best practices in ontology and vocabulary use [77], we started by considering reuse of classes and properties from existing vocabularies. We used the common set of properties we defined in the previous step, and found that the Schema.org vocabulary [59] was fully applicable for our set. The Schema.org vocabulary, as part of its Health and Lifesciences Extension [89], contains a definition of the class schema:Drug and contains a large set of properties applicable to it [90]. As we can see on Fig. 2, the RDF schema uses the DrugBank ontology and the RDFS ontology, as well, for interoperability purposes.

Schema.org is a joint initiative of Google, Bing, Yahoo and Yandex, as a common vocabulary intended for structured markup on web pages [91–93]. It is used by these search engines to introduce rich snippets about people, events, products, movies, restaurants, books, tv shows, etc. It is also used in Google’s Knowledge Graph, in emails confirming reservations and receipts (from restaurants, hotels, airlines, etc.) both from Gmail and Microsoft’s Cortana, it is used for rich pins on Pinterest, as well as from Apple’s Siri [94]. Its use on the Web has been increasing in the past few years, more rapidly than the more rigorous general-purpose vocabularies and ontologies before it [95]. Its success is mainly attributed to its simplicity: it uses a generally flat hierarchy of classes, so that the boundaries of implementation from data publishers and webmasters is kept low.

The growing use of the Schema.org vocabulary, as well as its domain generality, has put the vocabulary in a position in which it is being used for aligning existing ontologies and datasets. This is happening in the healthcare domain, as well [96]. With the release of Schema.org version 3.0 [97], the medical and healthcare related terms [98] have been moved to the Health and Livesciences Extension [89], to enable and ensure future collaborative development of the terms by the Healthcare Schema Vocabulary community group at W3C [99, 100]. This plan for a long-term support by the community from the domain instills sufficient certainty for us to choose the Schema.org vocabulary, instead of the domain specific ontologies [76], to provide a common schema for drug products on a global scale.

In order to provide some alignment between the generated datasets and the LODD and DrugBank datasets, we use several properties from the DrugBank ontology to describe the drug products. More specifically, we use drugbank:brandName, drugbank:genericName, drugbank:atcCode and drugbank:dosageForm as additional properties for the same values denoted by schema:name, schema:nonProprietaryName, schema:code and schema:dosageForm, respectively. We do this for simplifying the SPARQL federated queries when working with data from our LinkedDrugs dataset and the DrugBank dataset. Additionally, each drug product from the LinkedDrugs dataset is an instance of a specific class from the ATC Classification Ontology [101], in order to classify the drug according to the ATC classification system, based on its ATC code(s). We also chose rdfs:seeAlso as it is the most widely used relation for interlinking similar entities in the LOD Cloud [33].

Just as any other RDF schema, vocabulary and ontology, the RDF schema selected for our Linked Drug Data datasets can be evolve in time; it can be extended and modified in the future by us or third-parties, as the field of drug data evolves.

#### CSV template

In order to enable data publishers to annotate their drug data with the RDF schema from Fig. 2, we need a formal template for the data which is being prepared for transformation, and a formal transformation process. For the former, we define a CSV template, available publicly and as open-source [58]. The CSV template contains 39 columns which represent the different data fields needed from the source data for the transformation process. They inlude the URI of the drug, its brand name, generic name(s), manufacturer(s), ATC code(s), active ingredient(s), strength, cost, etc. They are modelled to fit with the RDF schema, which encompasses all data necessary for high-quality modeling of the domain.

The data type of the different columns is usually a simple text value, except where we note otherwise. Some important notes regarding the field data types include: the strength value is divided into an integer-value column denoting the strength, while the unit is part of a text-value column denoting the strenght unit; similarly, the cost of the drug is separated into a float value and a currency value; the several date columns need to be formatted as “YYYY-MM-DD”; the prescription status should be enumerated as either “OTC” or “PrescriptionOnly”; the currency code needs to comply with the ISO standard for denoting currencies [102]; the country where the drug is registered in needs to be denoted using a country code accoding to an ISO standard [63]; if there are multiple generic names, manufacturers or active ingredients, they should be denoted one-per-column in the available genericNameN, manufacturerN and activeIngredientN columns, respectively, etc. The details about the other column data types are available on the project website [58].

The CSV template uses a vertical line character (|) as a delimiter, since the regular CSV separators such as a comma (,) and a semicolon (;) are very often present in the cell values when working with drug data, and can be misinterpreted. It is important to note that the order of the columns in the CSV template is not relevant, if used with our OpenRefine transformation script.

As with the RDF schema, the CSV template is open and publicly available, and therefore can be extended or modified in the future by both us and third-parties, as the drug data field evolves and more Linked Drug Data dataset are being created.

#### OpenRefine transformation script

Step III of the methodology contains the task of transforming the source data into the RDF schema selected in Step II. Since we defined an RDF schema which can be applied in the drug data domain for drug products which are registered in different countries, we also provide a tool which can help automate the transformation process, while ensuring compliance of the generated data with the defined RDF schema and therefore providing aligned, high-quality 5-star Linked Data for the drug domain. The intent of this tool is to lower the bounds of transforming data into RDF and Linked Data for data publishers which are not deeply involved and experienced in the Semantic Web and Linked Data practices.

We provide this Linked Data generation tool in the form of an OpenRefine transformation script. OpenRefine [81] is an open-source software for working with structured data, usually CSV, TSV, XML, etc. It provides users with functionalities for working with large datasets: the users can record their action over a small set of example rows, and then apply them over the entire source data. Here, the actions can include data transformations, merging, data cleaning tasks, manipulation of the columns, etc. It also has an RDF extension which allows reconciliation of cell values against RDF data from SPARQL endpoints. This allows linking of cell values with entities from a SPARQL endpoint, for unambiguous identification of entities. It also allows mapping of the source data into RDF, by defining an ‘RDF skeleton’. The output of this action is an RDF file generated from the source data, according to the definitions in the ‘RDF skeleton’.

The ability of OpenRefine to save the user actions and then export them in JSON format, allows reuse of certain sets of actions for different datasets. This gives us the ability to define the data transformation which can be reused over different source drug datasets, which have the same columns. As we have a CSV template, we can use this as part as our set of tools. The defined list of data transformation actions we created is what we have as our OpenRefine transformation script [58].

Our OpenRefine transformation script is designed for data complying with the CSV template, and its output is a Linked Drug Data dataset which uses our RDF schema. The transformation script contains three actions:

A. reconcile the columns genericName1, genericName2,..., genericName5 against DBpedia,
B. reconcile the column atcCode against the DrugBank dataset, and
C. create an RDF schema skeleton

Action A. uses the RDF extension feature of OpenRefine which uses the cell value from a selected column to find potential entities from a given SPARQL endpoint which can be matched to the entity denoted by the row. In our case, we use the five genericName columns - which hold the generic name of the active ingredient of the drug entity - and we try to match each of them to a dbo:Drug entity from the DBpedia SPARQL endpoint, using its rdfs:label value. If the reconciliation service finds a matching candidate entity, we use it in step C. to create an RDF triple which links the drug entity from our CSV dataset with the matched candidates from DBpedia, via an rdfs:seeAlso relation, for instance:



Action B. does a similar reconciliation, but on the atcCode column from the CSV dataset and against the DrugBank endpoint. It tries to find matches between the value of the atcCode column on our side and the drugbank:atcCode value of drugbank:drugs instances from the endpoint. Unlike the situation in A., here we can have more than one matching candidate from DrugBank. The reason is that there can be multiple drugbank:drugs instances which have the same ATC code, i.e. share the same therapeutic, pharmacological and chemical properties. Similar as in A., we use all matching candidates from the reconciliation in step C. to create RDF triples which link the drug entity from our CSV dataset to the matched drug entities from DrugBank, such as:



Action C. creates the RDF schema skeleton, which contains the rules for mapping the consolidated CSV file into RDF. In the RDF schema skeleton (Fig. 2), we define mappings between the CSV columns and certain RDF triple patterns. Some of the mappings are straight-forward, such as the mappings of the brand name, the generic name, the dosage form, the country, the url, the description, etc. For them, we define the URI of the drug as a subject, we denote a specific property for the triple, and then we define the value of the column as a literal or an object of the triple. For instance, the brand name of a drug is mapped into RDF triples with the following format:



However, other mappings are more complex. Mappings of values such as the ATC code, the cost, the strength, the manufacturer, the license details, etc., need new entities to be created, entities of different types. For instance, in order to add the information about the ATC code to the drug entity, we need to create a new blank node of type schema:MedicalCode, which has two additional triples: one with the schema:codeValue property and one with the schema:codingSystem property. This ATC code mapping can be represented with:



The license mappings were the most complex, which we can see from Fig. 2. Aside from using OpenRefine’s user interface for defining the RDF skeleton, we used its GREL language for mapping the reconciliation results from actions A. and B. into rdfs:seeAlso triples.

As a result of the transformation script, a Linked Data dataset with links to the LOD Cloud is created. Similarly as the other tools, the transformation script is available as an open-source JSON file, which can be extended and modified in the future. As a support for it, we also developed a BASH script which sends the CSV file with drug data, formatted according to our CSV template, along with the OpenRefine transformation script to a running BatchRefine service [58]. The result from this call is the RDF output representing the transformed dataset.

#### SPARQL-based tool for extending and interlinking the dataset

Once the drug dataset is transformed into a Linked Data dataset with the other tools, an additional step is required in Step III to create the internal links between drugs which share the same functionality, i.e. share the same therapeutic, pharmacological and chemical properties, in order to create a better basis for use-cases. We need to create links between drugs from the dataset which have the same function, i.e. are aimed to treat the same condition. To create these links, we use drug’s ATC codes. According to the World Health Organization coding scheme [88], if two drugs have the same ATC code, they share the same function. For this purpose, we define a reusable SPARQL query [58] which detects all pairs of drugs from the dataset which have the same ATC code, and using the schema:relatedDrug property creates a pair of triples for them, for instance:



These two triples create a two-way link between the drugs in the dataset, denoting their functional similarity. The SPARQL query results with storing the newly created RDF triples in the same RDF graph where the dataset is already stored. These interlinkings can be utilizes for providing the users with alternative drugs they may require for treating their condition, either in the same of in a different country.

Since not all source registries contain the ATC code information, and in order to increase the number of interlinked drug products from the dataset and support better data analytics, we define an additional reusable SPARQL query [58] which assignes ATC codes to all drugs from the dataset which miss this information. The SPARQL query detects drug products without an ATC code, finds the generic drug from DBpedia which the drug is linked to with the rdfs:seeAlso relation, gets the ATC code of the DBpedia generic drug and assigns it to the drug product in question. Since the SPARQL query for interlinking drugs from the dataset depends on the ATC code, this SPARQL query for extending the dataset with missing ATC code values should be executed first.

Both SPARQL queries are parametrized and should be edited before execution. They can be executed over the Linked Data storage used for storing the Linked Data dataset generated with the other tools.

#### Web-based tool for automated transformation, interlinking and publishing

The generated Linked Drug Data dataset needs to be published on the Web according to the Linked Data principles and best practices, as advised in Step IV. In order to aid the data publishers, this step can be automatically executed by using a web-based tool we provide. The data publishers can upload the generated Linked Data dataset(s) on the LinkedDrugs project website [60], and after a human-based quality assessment, the dataset will be automatically published. For this we use a publicly available Virtuoso instance [61], from which the new dataset is available on the Web as Linked Data, via its SPARQL endpoint [62]. The RDF graph identifier is returned to the data publisher after the successful upload process.

Besides publishing finished Linked Drug Data datasets, the web-based tool and its automated process can also execute the previous steps of the methodology for the data publisher: (a) they can generate an interlinked Linked Data dataset from an input CSV file, and (b) they can interlink drugs with schema:relatedDrug relations from an input RDF file. For the former, the uploaded CSV file needs to be generated following our CSV template, and based on it, the predefined RDF schema and the OpenRefine transformation script, our web-based tool and its server-side process will generate the Linked Data dataset. Using the SPARQL-based tool from above, it will then generate links between the drugs from the dataset, based on their ATC codes. For the latter, the web-based tool directly creates the schema:relatedDrug relations between similar drugs from the uploaded Linked Drug Data dataset in RDF. With this, we provide the convenience to move most of the data processing from the methodological guidelines away from the data publishers, and simplify their workflow.

When a data publisher uses our web-based tool at [60] to publish a Linked Drug Data dataset, our system also adds it to the global Linked Drug Data dataset - the LinkedDrugs dataset - by storing it in another RDF graph and generating schema:relatedDrug triples for linking the drugs from the new dataset with the drugs from the existing datasets in LinkedDrugs, and vice-versa. The LinkedDrugs dataset then contains data for drug products provided by different publishers, including our team, and is available via a permanent, dereferenceable URI, which supports HTTP content negotiation [65].

## Endnote

^1^ Austria, Azerbaijan, Belgium, Canada, Costa Rica, Croatia, Cyprus, Czech Republic, Egypt, Estonia, EU’s European Medicines Agency, France, Hungary, Ireland, Italy, Macedonia, Malta, Netherlands, New Zealand, Nigeria, Norway, Romania, Russia, Serbia, Slovakia, Slovenia, South African Republic, Spain, Uganda, Ukraine and USA.
